# Glutamine-enriched enteral nutrition in very low birth weight infants. Design of a double-blind randomised controlled trial [ISRCTN73254583]

**DOI:** 10.1186/1471-2431-4-17

**Published:** 2004-09-01

**Authors:** Anemone van den Berg, Ruurd M van Elburg, Jos WR Twisk, Willem PF Fetter

**Affiliations:** 1 Department of Paediatrics, Division of Neonatology, VU University Medical Center, Amsterdam, the Netherlands; 2 Institute of Research in Extramural Medicine, VU University Medical Center, Amsterdam, the Netherlands

## Abstract

**Background:**

Enteral feeding of very low birth weight (VLBW) infants is a challenge, since metabolic demands are high and administration of enteral nutrition is limited by immaturity of the gastrointestinal tract. The amino acid glutamine plays an important role in maintaining functional integrity of the gut. In addition, glutamine is utilised at a high rate by cells of the immune system. In critically ill patients, glutamine is considered a conditionally essential amino acid. VLBW infants may be especially susceptible to glutamine depletion as nutritional supply of glutamine is limited in the first weeks after birth. Glutamine depletion has negative effects on functional integrity of the gut and leads to immunosuppression. This double-blind randomised controlled trial is designed to investigate the effect of glutamine-enriched enteral nutrition on feeding tolerance, infectious morbidity and short-term outcome in VLBW infants. Furthermore, an attempt is made to elucidate the role of glutamine in postnatal adaptation of the gut and modulation of the immune response.

**Methods:**

VLBW infants (gestational age <32 weeks and/or birth weight <1500 g) are randomly allocated to receive enteral glutamine supplementation (0.3 g/kg/day) or isonitrogenous placebo supplementation between day 3 and 30 of life. Primary outcome is time to full enteral feeding (defined as a feeding volume ≥ 120 mL/kg/day). Furthermore, incidence of serious infections and short-term outcome are evaluated. The effect of glutamine on postnatal adaptation of the gut is investigated by measuring intestinal permeability and determining faecal microflora. The role of glutamine in modulation of the immune response is investigated by determining plasma Th1/Th2 cytokine concentrations following in vitro whole blood stimulation.

## Background

Enteral feeding of very low birth weight (VLBW) infants is a challenge, since metabolic demands are high and administration of enteral nutrition is limited by immaturity of the gastrointestinal tract. In particular, small for gestational age VLBW infants may have impaired gut function, as fetal blood flow to heart, brain and adrenals is compensatory increased, while other organs including the gastro-intestinal tract are relatively hypoperfused in intrauterine growth retardation [1].

Experimental studies have shown that the amino acid glutamine plays an important role in maintaining functional integrity of the gut [2-5]. Glutamine serves as fuel for enterocytes [2] and provides nitrogen for the synthesis of amino sugars, involved in maintenance of tight junctions [3] and mucin synthesis [4]. Moreover, glutamine has a stimulatory and regulatory effect on mucosal cell proliferation and differentiation [5].

Glutamine is not only utilised at a high rate by intestinal epithelium but also by cells of the immune system. In vitro studies have shown that increasing availability of glutamine stimulates proliferation of T-lymphocytes in response to T-cell mitogens [6], phagocytosis and antigen presentation by monocytes [7] and Th1 cytokine response [8].

In critically ill patients, endogenous glutamine synthesis cannot meet increased demand and for this reason glutamine is considered a conditionally essential amino acid [9]. VLBW infants may be especially susceptible to glutamine depletion as placental supply suddenly ceases at birth, tolerance of enteral nutrition is limited and parenteral nutrition does not contain glutamine for solubility and stability reasons. Glutamine depletion has negative effects on the functional integrity of the gut [10] and leads to immunosuppression [11].

Studies in adults have shown that glutamine supplementation decreases mortality in critically ill adults [12], infectious morbidity in recipients of bone marrow transplantation [13] and multiple trauma patients [14] and length of hospital stay in surgical patients [15]. In VLBW infants, only two studies have investigated efficacy of glutamine supplementation [16,17]. In the study of Lacey *et al *[16], 78 VLBW infants at high risk of developing necrotising enterocolitis (birth weight 530–1250 g) were randomised to receive either standard or glutamine-supplemented parenteral nutrition. After exclusion of 34 infants, 22 treated and 22 control infants were compared for length of stay, days on total parenteral nutrition, days on the ventilator and infectious morbidity. In infants with a birth weight ≥ 800 g no effect of glutamine supplementation was found. However, in infants with a birth weight <800 g glutamine supplementation was associated with shorter time to full enteral nutrition, fewer days on parenteral nutrition, fewer days on ventilatory support and reduced length of stay. Incidence of positive blood cultures and rate of weight gain were not different in glutamine and control groups. Neu *et al *[17] performed a randomised controlled trial of glutamine-enriched enteral nutrition in 68 VLBW infants with a gestational age of 24–32 weeks and a birth weight of 500–1250 g. Analysis was performed in 66 infants on an intention to treat basis. Feeding tolerance (as measured by number of days on which feeding had to be withheld) was better in the glutamine group compared to the control group. In addition, after adjusting for birth weight the odds ratio of developing sepsis was 3.8 for the control group compared to the glutamine group. Average weight at different time intervals and length of stay were not different between the groups. Although some methodological concerns can be raised (the sample size of both studies is small; Lacey *et al *[16] did not perform analysis on an intention to treat basis), these studies suggest that glutamine supplementation enhances feeding tolerance and decreases infectious morbidity in VLBW infants.

The current double-blind randomised controlled trial is designed to determine the effect of glutamine-enriched enteral nutrition on feeding tolerance in a sufficient large population VLBW infants. We hypothesise that time to full enteral feeding is shorter in infants who receive glutamine-enriched nutrition compared to infants in the control group. Furthermore, infectious morbidity and short-term outcome are evaluated.

To elucidate the effect of glutamine-enriched enteral nutrition on the functional integrity of the gut, intestinal permeability for macromolecules is measured. As part of the postnatal adaptation of the gut, the intestinal permeability decreases during the first days of life [18]. We hypothesise that glutamine-enriched enteral nutrition stimulates postnatal adaptation of the gut, reflected by a larger decrease in intestinal permeability. Another aspect of postnatal adaptation of the gut is the development of intestinal microflora. In VLBW infants, the colonisation by bacteria (including beneficial *Bifidobacterium *and *Lactobacillus *species [19,20]) commonly present in healthy breast fed infants is delayed [21]. Intestinal mucin is an important site for bacterial adhesion and colonisation [22]. Glutamine may improve mucin quality [4] and consequently influence bacterial colonisation. We hypothesise that glutamine-enriched enteral nutrition stimulates the presence of *Bifidobacterium *and *Lactobacillus *species in the intestinal microflora. Intestinal microflora is investigated by determining faecal microflora with a culture independent technique.

The effect of glutamine-enriched enteral nutrition on the immune response is investigated by determining plasma T-helper type 1 (Th1) and T-helper type 2 (Th2) cytokine concentrations following in vitro whole blood stimulation. As pregnancy is associated with skewing towards Th2 immunity [23], Th2 cytokine responses dominate the neonatal immune response [24]. Exposure to microbes stimulates Th1 cytokine responses and deviates the neonatal immune response towards balanced Th1/Th2 cytokine responses [24]. We hypothesise that glutamine-enriched enteral nutrition contributes to balanced Th1/Th2 cytokine responses by stimulating the Th1 cytokine response [8].

To assess safety of glutamine-enriched enteral nutrition, plasma amino acid profiles are determined. We hypothesise that plasma amino acid profiles in glutamine and control groups will not differ during the study period. In addition, to exclude negative effects of glutamine-enriched enteral nutrition on neurodevelopmental outcome, neuromotor development at the corrected age of 1 and 2 years and mental/motor development at the corrected age of 2 years are assessed.

In conclusion, this double-blind randomised controlled trial aims to determine the effect of glutamine-enriched enteral nutrition on feeding tolerance, infectious morbidity and short-term outcome in VLBW infants. In addition, an attempt is made to elucidate the role of glutamine in postnatal adaptation of the gut and modulation of the immune response.

## Methods

The study is designed as a double-blind randomised clinical trial. The national central committee on research involving human subjects and the medical ethical review board of our hospital approved the study protocol.

### Study population

Infants with a gestational age <32 weeks and/or birth weight <1500 g admitted to the level III neonatal intensive care unit (NICU) of the VU University Medical Center, Amsterdam, are eligible for participation in the study. Written informed consent is obtained from all parents.

Exclusion criteria are: major congenital or chromosomal anomalies, death <48 h after birth, transfer to another hospital <48 h after birth and admission from an extraregional hospital.

### Treatment allocation and blinding

To balance birth weight distribution into treatment groups, each infant is stratified to one of three birth weight groups (<799 g, 800–1199 g, ≥ 1200 g) and randomly allocated to treatment <48 hours after birth. An independent researcher uses a computer-generated randomisation table based on blocks of four (provided by Nutricia Nederland BV, Zoetermeer, The Netherlands) to assign infants to treatment A or B, which correspond to batch numbers on the nutrition products. Investigators, parents, medical and nursing staff are unaware of treatment allocation. The code for the batch numbers is broken after data analysis is performed.

### Treatment

Glutamine powder contains 82% L-glutamine and 18% glucose (nitrogen 15.5 wt/wt%; 371 kcal/100 g), whereas the isonitrogenous control powder contains 100% L-alanine (nitrogen 15.7 wt/wt%, 435 kcal/100 g). The two powders are indistinguishable by appearance, colour and smell. During the study period, glutamine and control powder are monitored for stability and microbiological contamination.

Between days 3 and 30 of life, supplementation is administered in increasing doses to a maximum of 0.3 g/kg glutamine per day in the glutamine group. Initially, the supplementation dose is based on birth weight. After 2 weeks the dose is adjusted to actual weight. Two members of the nursing staff daily add supplementation to breast milk or to preterm formula (Nenatal^®^, Nutricia Nederland B.V., Zoetermeer, The Netherlands), according to the parents' choice. Per 100 ml, Nenatal^® ^provides 78 kcal, 2.1 g protein (casein-whey protein ratio 40:60), 4.4 g fat and 7.5 g carbohydrate. Nenatal^® ^does not contain free L-glutamine. When infants are transferred to other hospitals before the end of the study, the protocol is continued under supervision of the principal investigator.

### Nutritional support

Protocol guidelines for the introduction of parenteral and enteral nutrition follow current practice at our NICU. Administration of parenteral nutrition starts at day 2 and will be advanced gradually until amino-acid intake reaches 3 g/kg/day at day 6. Parenteral nutrition is discontinued if enteral feeding reaches a volume of approximately 150 mL/kg/day. Parenteral nutrition, an all-in-one mixture provided by the hospital pharmacy, contains per 100 mL 54 kcal, 8.5 g glucose, 1.7 g amino acids and 1.7 g lipids. If necessary, glucose, amino acids and lipids are given in separate solutions.

Guidelines for the introduction of enteral nutrition are as follows: 1. minimal enteral nutrition starts at day 1 (6–12 mL daily); 2. enteral nutrition is advanced either from day 3 or from day 5 in case of complications: BW <p10, GA <26 weeks, Apgar score at 5 minutes <6, umbilical artery pH <7.10 or base deficit >10 mmol/L; 3. feeding is advanced at a dose of 15–20 mL/kg/day to a maximum of 150 mL/kg/day (based on actual weight). Furthermore, guidelines for reduction/withholding of enteral feeding are: 1. enteral feeding is reduced/withheld in case of gastric residuals (> total volume of past 2 feedings), bilious residuals, emesis, ileus or necrotising enterocolitis Bell's stage ≥ II [25]; 2. when signs of feeding intolerance resolve, feeding is advanced in the volume given before reduction/withholding within 2 days.

For each infant in the study a feeding schedule is proposed, based on birth weight and the guidelines as mentioned above. However, the staff of our NICU has final responsibility for the administration of parenteral nutrition and advancement of enteral feeding.

### Study outcome measures

#### Study outcome measures

Primary outcome of the study is time to full enteral feeding, defined as a feeding volume ≥ 120 mL/kg/day. Furthermore, other parameters of feeding tolerance, infectious morbidity, and short-term outcome are evaluated (Table 1). In addition to clinical outcome, intestinal permeability, faecal flora, plasma Th1/Th2 cytokine concentrations and plasma amino acid profiles are determined during the 30 day study period (Table 2).

**Table 1:** Clinical outcome measures

	**Remarks**
	
**Feeding tolerance**	
Enteral feeding >120 mL/kg/day	Primary outcome
Age at finishing parenteral nutrition	
Days of no enteral feeding during study period	
Necrotising enterocolitis	Bell *et al *[25]
**Infectious morbidity**	
Serious infections	
Number of infectious episodes	
Cultured micro-organisms	
**Short-term outcome**	
Weight *z *scores at birth, day 30 and at discharge	Usher *et al *[33]
Patent ductus arteriosus	
Ventilatory support	
Use of oxygen at postmenstrual age of 36 weeks	Jobe *et al *[34]
Intraventricular hemorrhage	Papile *et al *[35]
Retinopathy of prematurity	Committee for ROP [36]
Death	
Age at discharge from NICU and at discharge home	

ROP = retinopathy of prematurity; NICU = neonatal intensive care unit.

**Table 2:** Study schedule

	**< 48 h**	**day 7**	**day 14**	**day 30**
				
Amino acid profile	x	x	x	x
Intestinal permeability	x	x	x	x
Faecal flora	x	x	x	x
Th1/Th2 cytokine profile	x	x	x	-

#### Clinical outcome measures

The following perinatal characteristics are registered to assess prognostic similarity: maternal age and race, obstetric diagnosis, administration of antenatal steroids and antibiotics, mode of delivery, sex, gestational age, birth weight, birth weight <p10, Apgar scores, pH of the umbilical artery, clinical risk index for babies [26] and administration of surfactant.

During the study period actual intake of enteral and parenteral nutrition, powder supplementation and type of feeding (breast milk or preterm formula) are recorded daily.

Evaluation of medical records for the presence of serious infections is performed by one investigator/neonatologist, unaware of treatment allocation. Serious infections include sepsis, meningitis, pyelonephritis, pneumonia, and arthritis. Sepsis work-up consists of blood, cerebrospinal fluid and urine (suprapubic bladder tap) culture. Sepsis is defined as the combination of a positive blood culture and the presence of at least two clinical signs (body temperature <36.5°C or >37.5°C, hypotension, tachycardia, apnoeic attacks, feeding problems, irritability or apathy). Meningitis is diagnosed when micro-organisms are cultured in the cerebrospinal fluid. Pyelonephritis is diagnosed when both urine culture and dimercaptosuccinic acid (DMSA) renal scan are positive. Pneumonia is defined as the combination of a positive culture of tracheal aspirate, bronchial secretion or sputum and the presence of at least one clinical sign in ventilated infants (purulent sputum, changed sputum characteristics or deterioration of ventilation settings) or at least two clinical signs in non-ventilated infants (tachypnea, cyanosis, wheezing/rales/crepitation or purulent sputum/changed sputum characteristics). Arthritis is defined as the combination of a positive culture of intra-articular fluid and the presence of signs of articular inflammation.

#### Postnatal adaptation of the gut

The effect of glutamine-enriched enteral nutrition on postnatal adaptation of the gut is studied by measuring intestinal permeability and by determining faecal flora.

Intestinal permeability is measured by the sugar absorption test, as previously described [18]. After instillation of the test solution, 2 ml/kg by nasogastric tube, urine is collected for 6 hours. After collection, 0.5 ml chlorohexidine digluconate 20% (preservative) is added to the urine and samples are stored at -20°C until analysis. Lactulose and mannitol concentrations (mmol/mol creatinine) are measured by gas chromatography as previously described [27]. The lactulose/mannitol ratio is used as a measure of intestinal permeability.

Faecal samples are stored at -20°C until analysis by fluorescent in situ hybridisation (FISH) using specific 16S rDNA-targeted probes as described by Harmsen *et al *[28].

#### Immune response

The effect of glutamine enriched-enteral nutrition on the immune response is investigated by determining plasma Th1/Th2 cytokine concentrations following in vitro whole blood stimulation. Heparinized blood (0.5 mL), diluted 1:1 in sterile medium (RPMI 1640 without L-glutamine, Gibco, Paisley, United Kingdom) is stimulated for 24 h at 37°C in the presence of anti-CD3/anti-CD28 (Central Laboratory of the Netherlands Red Cross Blood Transfusion service, Amsterdam, the Netherlands) and *Escherichia coli *lipopolysaccharide (concentration 1:1000 both). After incubation, blood is centrifugated, supernatant is collected and stored at -20°C until analysis. Th1 cytokines IFN-γ, TNF-α, IL-2 and Th2 cytokines IL-4, IL-5 and IL-10 are measured by cytometric bead array (BD biosciences, Alphen aan den Rijn, the Netherlands).

#### Safety

Safety of enteral glutamine supplementation in a dosage of 0.3 g/kg/day is investigated by determination of plasma amino acid profiles. Immediately after withdrawal, heparinized blood (0.5 mL) is centrifuged at 10000 rpm for 4 minutes. Plasma is deproteinized by sulfosalicylic acid (2mg/100 μL) and stored at -70°C until analysis. Amino acid profiles are determined by high-performance liquid chromatography as described by Teerlink *et al *[29].

To investigate neurodevelopmental outcome, neuromotor development at the corrected age of 1 and 2 years [30] and mental/motor development at the corrected age of 2 years are assessed [31].

### Sample size

We have calculated that a sample size of 102 infants is necessary to detect a difference of at least 2.5 days in time to full enteral feeding, assuming a SD of 4.5 days (two-tailed α = 0.05, β = 0.20). The SD value is based on an retrospective analysis of time to full enteral feeding in infants with GA <32 weeks and/or BW <1500 g admitted to our NICU in 1998.

### Statistical analysis

To determine whether randomisation is successful, prognostic similarity (perinatal and nutritional characteristics) between treatment groups is assessed. The Students' t-test, Mann-Whitney U test, and chi-square test or Fisher's exact test are used to compare continuous normally distributed data, nonparametric continuous data and dichotomous data respectively.

Cox regression is performed to examine the effect of glutamine-enriched enteral nutrition on time to full enteral feeding. Logistic regression is performed to examine whether glutamine-enriched enteral nutrition influences the incidence of serious infections. In an additional analysis, adjustments are made for possible confounding factors as administration of antenatal corticosteroids, birth weight <p10, administration of breast milk and other prognostic factors that may be different between treatment groups.

Analyses of secondary outcomes (only crude) is performed by Mann-Whitney U test, chi-square test or Fisher's exact test and log rank test for nonparametric continuous data, dichotomous data, and time-dependent data respectively.

Generalised estimated equations for longitudinal analysis [32] are used to analyse changes over time in intestinal permeability, faecal microflora, plasma Th1/Th2 cytokine concentrations and plasma amino acid profiles.

Distribution of optimal and non-optimal neuromotor development and normal and abnormal mental/motor development in glutamine and control groups is examined by logistic regression with adjustments for possible confounding factors as gestational age and birth weight.

All statistical analyses are performed on an intention to treat basis. In addition, alternative per protocol analyses are performed, excluding all patients who are not treated according to protocol, defined as more than 3 consecutive days or a total of 5 days on minimal enteral feeding or without supplementation.

A p value <0.05 is considered significant (two-tailed). SPSS 9.0 (SPSS Inc., Chicago, IL, USA) and STAT 7.0 (StatCorp LP, College Station, TX, USA) are used for data analysis.

## Competing interests

Nutricia Nederland B.V. (Zoetermeer, the Netherlands) provided Nenatal^®^, glutamine and placebo supplementation.

## Authors' contributions

Ruurd van Elburg and Willem Fetter formulated the research question and wrote the study protocol. Anemone van den Berg, Ruurd van Elburg and Willem Fetter contributed to the development of the protocol. Jos Twisk gave advice on data analysis. Anemone van den Berg wrote the draft for this manuscript and the other authors reviewed the manuscript. All authors approved the final version of the manuscript.

## Pre-publication history

The pre-publication history for this paper can be accessed here:


